# Laparoscopic versus open major liver resection for hepatocellular carcinoma: systematic review and meta-analysis of comparative cohort studies

**DOI:** 10.1186/s12885-019-6240-x

**Published:** 2019-11-06

**Authors:** Zi-Yu Wang, Qing-Lian Chen, Ling-Ling Sun, Shu-Ping He, Xiao-Fen Luo, Li-Shuang Huang, Jun-Hai Huang, Cheng-Ming Xiong, Chong Zhong

**Affiliations:** 1 Department of Hepatobiliary Surgery, the First Affiliated Hospital of Guangzhou University of Chinese Medicine, 16 Airport Road, Guangzhou, 510405 China; 2 Lingnan Medical Research Center, Guangzhou University of Chinese Medicine, Guangzhou, 510405 China; 3 The First Clinical Medical School of Guangzhou University of Chinese Medicine, Guangzhou, 510405 China; 4 Department of Oncology, the First Affiliated Hospital of Guangzhou University of Chinese Medicine, Guangzhou, 510405 China; 5 School of Nursing, Guangzhou University of Chinese Medicine, Guangzhou, 510405 China

**Keywords:** Laparoscopic surgery, Major liver resection, Hepatocellular carcinoma, Meta-analysis

## Abstract

**Background:**

The application of laparoscopic liver resection (LLR) has expanded rapidly in recent decades. Although multiple authors have reported LLR shows improved safety and efficacy in treating hepatocellular carcinoma (HCC) compared with open liver resection (OLR), laparoscopic (LMLR) and open (OMLR) major liver resections for HCC treatment remain inadequately evaluated. This work aimed to test the hypothesis that LMLR is safer and more effective than OMLR for HCC.

**Methods:**

Comparative cohort and registry studies on LMLR and OMLR, searched in PubMed, the Science Citation Index, EMBASE, and the Cochrane Library, and published before March 31, 2018, were collected systematically and meta-analyzed. Fixed- and random-effects models were employed for generating pooled estimates. Heterogeneity was assessed by the Q-statistic.

**Results:**

Nine studies (1173 patients) were included. Although the pooled data showed operation time was markedly increased for LMLR in comparison with OMLR (weighted mean difference [WMD] 74.1, 95% CI 35.1 to 113.1, *P* = 0.0002), blood loss was reduced (WMD = − 107.4, 95% CI − 179.0 to − 35.7, *P* = 0.003), postoperative morbidity was lower (odds ratio [OR] 0.47, 95% CI 0.35 to 0.63, *P* <  0.0001), and hospital stay was shorter (WMD = − 3.27, 95% CI − 4.72 to − 1.81, *P* <  0.0001) in the LMLR group. Although 1-year disease-free survival (DFS) was increased in patients administered LMLR (OR = 1.55, 95% CI 1.04 to 2.31, *P* = 0.03), other 1-, 3-, and 5-year survival outcomes (overall survival [OS] and/or DFS) were comparable in both groups.

**Conclusions:**

Compared with OMLR, LMLR has short-term clinical advantages, including reduced blood loss, lower postsurgical morbidity, and shorter hospital stay in HCC, despite its longer operative time. Long-term oncological outcomes were comparable in both groups.

## Background

Hepatocellular carcinoma (HCC) represents the second deadliest malignancy around the world [1]. HCC is the unique type of malignancy for which the mortality rate continues to rise despite impressive advances in anticancer treatment [2]. Hepatic resection remains an essential treatment strategy for HCC. The initial application of laparoscopic liver resection (LLR) was in 1991 [3]. From then on, due to technological development and the improvement of laparoscopic liver surgery, the number of LLRs has increased dramatically in the past quarter-century [4]; indeed, LLR has been regarded as a great advance in modern liver surgery [5–9]. LLR can be divided into two categories, i.e., (i) laparoscopic minor liver resection, which involves non-anatomic wedge resection, left lateral resections, and/or removal of anterior liver segments (4b, 5, 6), and (ii) laparoscopic major liver resection (LMLR), which includes removal of right and left hepatic hemispheres, trisectionectomy, and resection of posterior segments (1, 4a, 7, 8) [10]. Although surgical and oncological outcomes, including peri- and post-operative outcomes, overall survival (OS) and disease-free survival (DFS), are considered to be similar for LLR and open liver resection (OLR) in HCC, most studies only described laparoscopic minor liver resection [11–15]. Ciria et al. [8] conducted one of the largest reviews of LLR compared with OLR, and suggested LLR might provide ameliorated short-term outcomes. However, the above study only focused on surgical outcomes and did not compare oncological outcomes. As technological support advances and experience in minimally invasive surgery grows, LLR has been developed from minor to major resection for the treatment of HCC [16, 17]. LMLR is increasingly practiced in high-volume and specialized centers. However, its application requires further evaluation. Therefore, an update on the worldwide situation is necessary to assess the current status of LMLR, especially focusing on its advantages and drawbacks comparatively to open major liver resection (OMLR).

Obviously, a prospective, randomized trial would be ideal for assessing the surgical and oncological outcomes of LMLR versus OMLR. Actually, according to the records of Clinicaltrial.org, several prospective, randomized control trials on LLR versus OLR have been carried out, including NCT02014025, NCT01768741, NCT02526043, NCT00606385, NCT02014025, and NCT02131441. However, to our knowledge, no reports are currently available on these randomized controlled trials (RCTs).

Therefore, the current work primarily aimed to perform a systematic review of the global clinical evidence of LMLR versus OMLR for HCC by assessing reports published before March 2018. These reports were meta-analyzed to investigate perioperative and postoperative surgical outcomes as well as long-term oncological outcomes, comparing LMLR and OMLR.

## Methods

The methods used in this study included a literature search. Eligibility criteria for studies, outcome measures, and statistical analyses followed the protocol recommended by Stroup et al., and Shamseer et al [18, 19]

### Data sources and searches

Studies published in PubMed, EMBASE, the Science Citation Index, the Cochrane Library, and secondary databases, were reviewed as the primary sources. The time of publication was restricted from January 1, 1991 to March 31, 2018. A PubMed query was performed with (“Carcinoma, Hepatocellular/mortality”[Majr]) AND (laparoscopy OR laparoscopic OR minimally invasive OR liver resection OR hepatectomy) AND (major liver resection OR major hepatectomy OR posterior segment OR hemihepatectomy OR trisectionectomy). EMBASE and the Science Citation Index were searched with [(‘laparoscopy’/mj OR laparoscopic) AND (‘liver’/mj AND ‘resection’/mj OR ‘hepatectomy’/mj) AND (liver AND cancer OR (liver AND tumor) OR (hepatocellular AND carcinoma))]. The search was extended to “related articles” to obtain additional interesting articles. We also manually searched for interesting references listed in the retrieved articles. In case two or more studies were published by the same authors or institution, the most recently reported trial or the one of highest quality was selected.

### Study selection

RCTs comparatively assessing LMLR and OMLR in HCC for peri- and post-operative surgical parameters and/or long-term oncological outcomes (OS and DFS) were reviewed. Criteria for LMLR were defined in accordance with previously described guidelines [10]. Inclusion criteria were: (1) confirmed HCC diagnosis; (2) patients with no contraindication for LLR; (3) a pure laparoscopic approach performed, without any additional procedures; (4) LLR or OLR procedures for hemihepatectomy, trisectionectomy, and resection of difficult posterior segments (4a, 7, 8, 1), considered major liver resections; and (5) full-length articles of studies in which ≥20 patients were evaluated.

Exclusion criteria were: non-human or experimental studies; non-research-based articles, such as reviews, editorials, letters, and case reports; studies including less than 20 patients; publications on LMLR for recurrent HCC, hepatic metastatic cancer, or simultaneous resection of liver and other organs; studies reporting simultaneous malignant and benign liver tumors, learning curves for surgical techniques, or lacking OLR data; reports on hand-assisted laparoscopic resection; articles only reporting minor liver resections or with the outcomes of major liver resection unavailable for assessment.

### Outcomes assessment

The outcomes assessed involved perioperative (operative time, blood loss, blood transfusion, and surgery margin), postoperative (negative rate of surgical margin/R0 resection, postoperative morbidity, and hospital stay duration) and long-term oncological (1-, 3-, and 5-year OS and DFS) outcomes. By definition, surgical margin means the margin of seemingly non-cancerous tissue surrounding a surgically resected tumor; R0 and R1 hepatectomies mean no (negative surgery margin) and some (microscopic positive margin) malignant cells observed by microscopy at the resection margin, respectively. Other outcomes involved in the included articles were reviewed simultaneously. The primary aim of this work was to provide a perspective on the worldwide status of LMLR by systematically reviewing comparative studies that reported LMLR and OMLR outcomes. Therefore, we performed a meta-analysis evaluating (i) the perioperative and postoperative surgical outcomes and (ii) the long-term oncological outcomes of LMLR versus OMLR in comparative cohort studies.

### Data and quality assessment

CZ conceived and designed the study. Two reviewers (ZYW and QLC) independently evaluated potentially eligible studies, taking into account their titles, abstracts, and full texts. In case of disagreement regarding the eligibility of a study, its full text was downloaded for further assessment. Data extraction was carried out by both reviewers (CZ and LLS) independently; SPH, XFL, LSH, JHH, and CMX analyzed and interpreted the data; ZYW and CZ wrote and revised the manuscript. The quality of included articles was evaluated as previously described [20].

### Data analysis and synthesis

Odds ratios (ORs) and weighted mean differences (WMDs) with 95% confidence intervals (CIs) were employed for evaluation in this study. When means were not reported in the included studies, they were estimated using the median, range, and sample size according to a method recommended by Hozo et al [21] Heterogeneity was deemed non-statistically significant with *P* > 0.1 as assessed by the Cochran Q test. In this case, the fixed-effects model was utilized for the meta-analysis. In case of heterogeneity, the random-effects model was used instead. Variances were employed for assessing the weights of various studies. Effect size consistency was assessed by the I^2^ statistic. I^2^ values below 25%, from 25 to 50%, and above 50% were considered to represent low heterogeneity, moderate heterogeneity, and high heterogeneity, respectively [22].

## Results

### Eligible studies and worldwide descriptive statistics

**Fig. 1:**
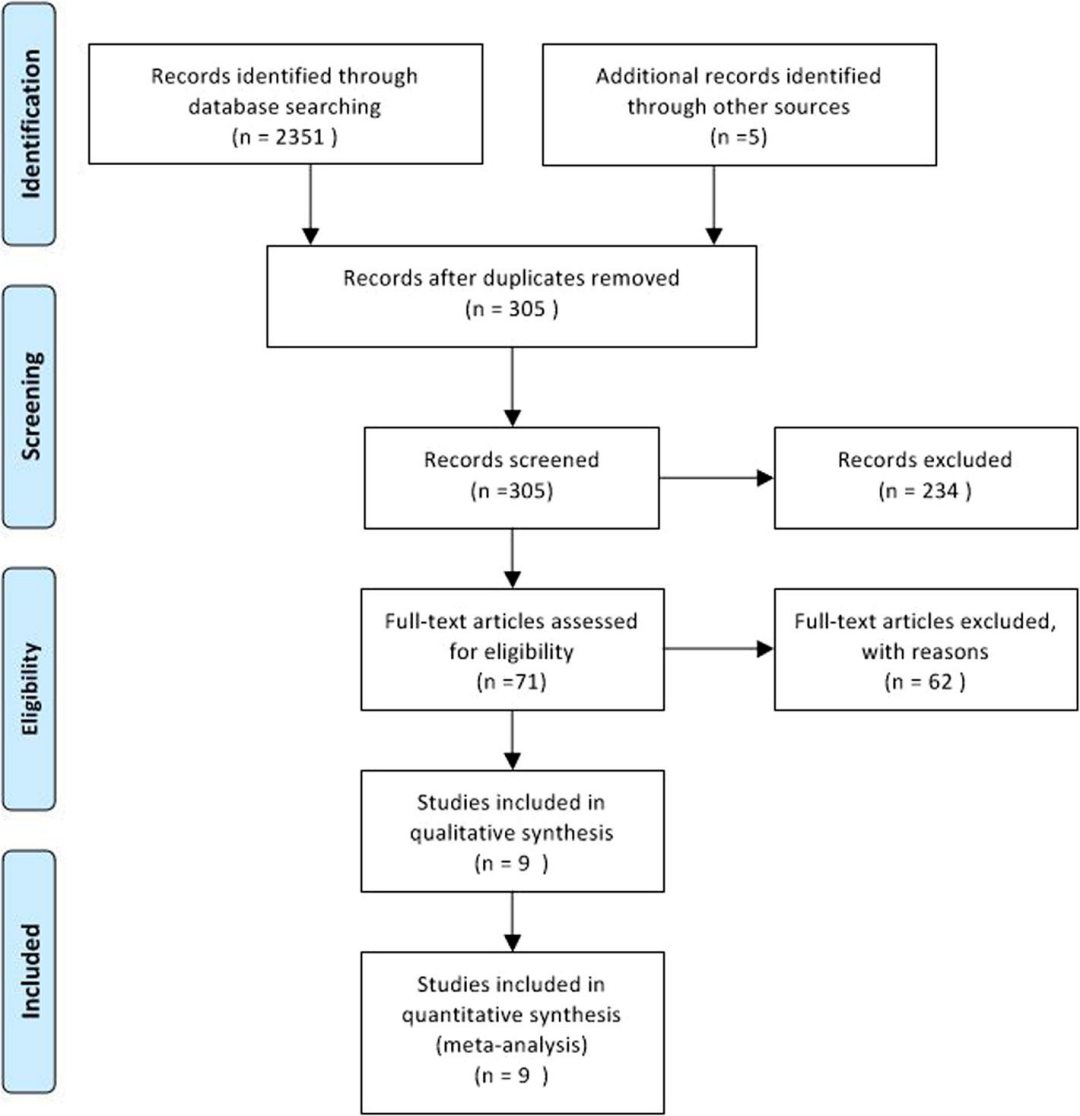
Flow chart of article screening and meta-analyses performed in this study

Figure 1 illustrates the study screening and review processes. The detailed features of the included articles are listed in Table 1. In all, 1173 patients (LMLR 447, OMLR 726) from 9 reports were assessed [23–31]. All included trials were single-center retrospective studies with comparable demographics and tumor features in both groups. Patient number per trial was between 43 and 259. The patients included 951 men and 222 women. Patients underwent LMLR or OMLR following clinical HCC diagnostic, based on serum alpha-fetoprotein amounts, liver function, preoperative three-phase multislice computed tomography (CT), and/or magnetic resonance imaging (MRI). HCC confirmation was performed by pathology.

**Table 1:** Characteristics of Studies Included

Reference/Country/Journal (year)	Study Period (year)	Study type	Sample Size (n, y)		Sex ratio (M/F)		Cirrhosis (y/n), or ICGR_15_	
			LMLR	OMLR	LMLR	OMLR	LMLR	OMLR
Cho JY/Korea/ Surgery(2015) [23]	2003–2012	R	24 (53.9 ± 12.6)	19 (60.0 ± 8.9)	17/7	16/3	10/24 ICG 8.2 ± 7.3	N/A ICG 6.4 ± 4.2
Xiao L/China/Surg Endosc (2015) [24]	2010–2012	RM	41 (52.07 ± 11.62)	86 (50.28 ± 11.89)	34/7	77/9	33/41	72/86
Komatsu S/France/Surg Endosc (2016) [25]	2000–2014	RM	38 (61.5 ± 12.2)	38 (61.7 ± 16.1)	34/4	33/5	31/7	28/10
Zhang Y/China/Surg Laparosc Endosc Percutan Tech (2016) [26]	2012–2014	RM	20 (47 ± 8.5)	25 (52 ± 10.5)	12/8	15/10	20/0	25/0
Chen JH/China/Medicine (2017) [27]	2015–2016	RM	126 (51, 21–76)	133 (51, 12–74)	93/33	108/25	ICGR_15_ 4.8 ± 3.8	ICGR_15_ 4.3 ± 4.8
Yoon YI/Korea/Ann Surg (2017) [28]	2007–2015	RM	37 (55.19 ± 7.12)	115 (58.37 ± 9.89)	26/11	93/22	ICGR_15_ 11.6 ± 4.72	ICGR_15_ 13.67 ± 5.51
Guro H/Korea/Surg Oncol (2018) [29]	2004–2015	RM	67 (57.7 ± 11.1)	110 (59.11 ± 12.3)	49/18	93/17	ICGR_15_ 9.1 ± 8.3	ICGR_15_ 9.5 ± 5.9
Rhu J/Korea/World J Surg (2018) [30]	2009–2016	RM	58 (58.2 ± 8.8)	133 (57.9 ± 9.7)	46/12	114/19	ICGR_15_ 11.7 ± 5.4	ICGR_15_ 11.0 ± 4.0
Xu H/China/Surg Endosc (2018) [31]	2015–2017	RM	36 (53.5 ± 11.0)	67 (49.0 ± 13)	30/6	61/6	ICGR_15_ 4.8 ± 2.2	ICGR_15_ 4.9 ± 2.1

Variables are expressed as mean ± SD or no. (%), unless otherwise indicated

*Abbreviations*: *LMLR* laparoscopic major liver resection, *OMLR* open major liver resection, *ICGR*_*15*_ indocyanine green retention rate at 15 min, *R* retrospective, *RM* retrospective matched

### Quality of included studies

Study quality and risk of bias were evaluated by the modified Newcastle–Ottawa scale (NOS) (Table 2). The included cohort trials all had moderate quality (NOS score ≤ 6). All full-length articles of the included studies were downloaded for assessment. The trials were retrospective or retrospective matched single-center studies reported between January 2015 and March 2018. The LMLR and OMLR groups were compared solely for major liver resection. Although the surgical and oncological outcomes were possibly affected by selection bias in three included studies, the propensity score matching method was applied to minimize the bias [28, 30, 31]. However, how missing data were handled was not fully disclosed in most included reports.

**Table 2:** Methodological Assessment

Reference	Selection				Comparability	Outcome			Total Points
	Representativeness	Selection	Ascertainment	Conflicted Interest	Comparability	Assessment	FU Length	Adequacy of FU	
Cho JY [23]	1	1	1	1	1	0	1	0	6
Xiao L [24]	1	1	1	1	1	0	0	0	5
Komatsu S [ 25]	1	1	1	1	1	1	0	0	6
Zhang Y [26]	1	1	1	1	1	1	0	0	6
Chen J [27]	1	1	1	1	1	0	0	0	5
Yoon YI [28]	1	1	1	1	1	1	0	0	6
Guro H [29]	1	1	1	1	1	1	0	0	6
Rhu J [ 30]	1	1	1	1	1	1	0	0	6
Xu H [31]	1	1	1	1	1	1	0	0	6

1 = consistent with criteria and low risk of bias; 0 = not consistent with criteria and high risk of bias. *N/A* indicates not applicable, *FU* follow-up

A maximum of 2 points can be achieved for this criterion

### Perioperative outcomes

**Fig. 2:**
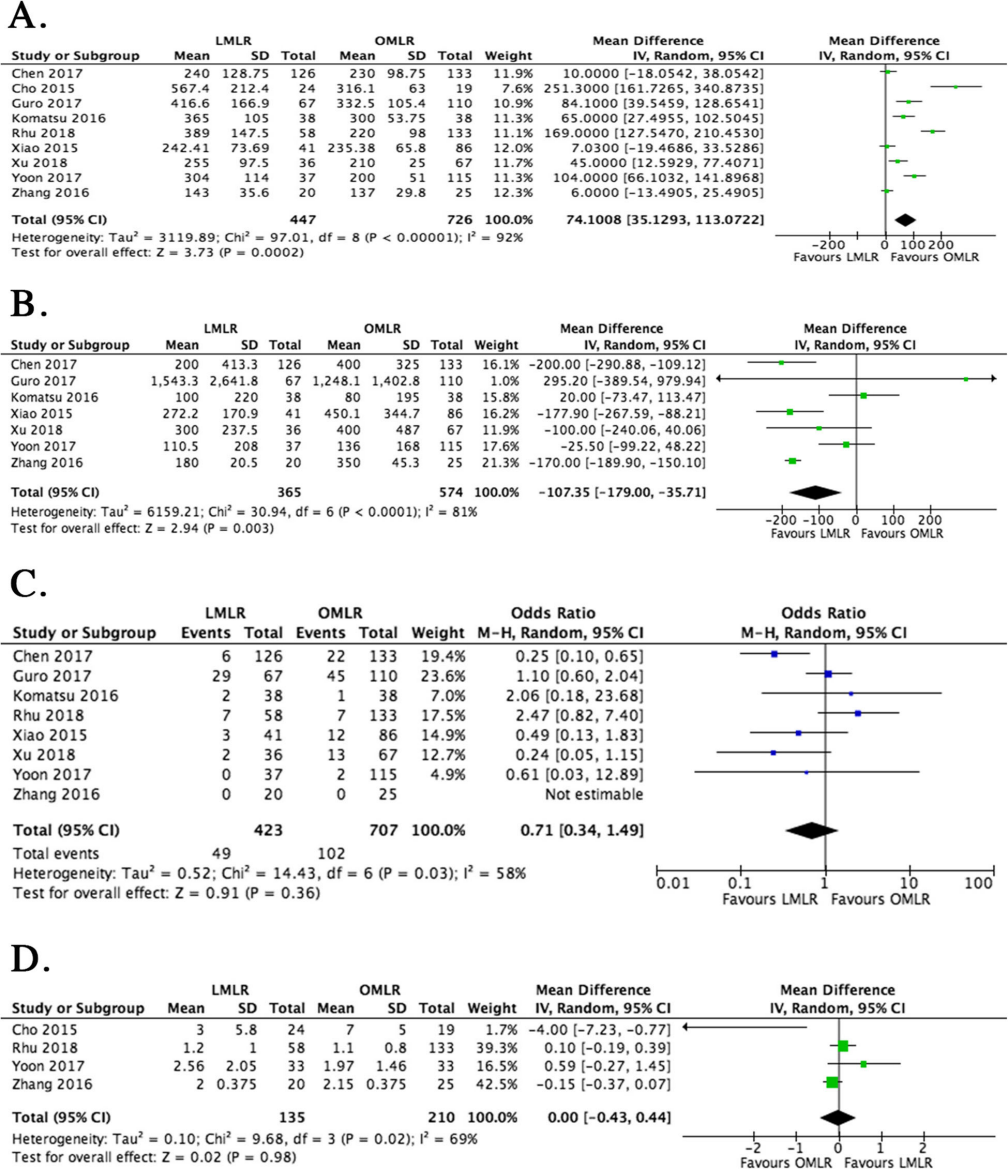
Forest plots depicting perioperative outcomes of LMLR versus OMLR. **a**. Operative time of LMLR versus OMLR; **b.** Blood loss in LMLR versus OMLR; **c.** Blood transfusion in LMLR versus OMLR; **d**. Resection margin in LMLR versus OMLR. Weighted mean differences (WMDs) and Odds ratios (ORs) are shown with 95% confidence intervals (CIs). LMLR, laparoscopic major liver resection; OMLR, open major liver resection

Perioperative outcomes were summarized as follows. The operative time was starkly prolonged in the LMLR group compared with the OMLR group (WMD = 74.1, 95%CI 35.1 to 113.1 min, *P* = 0.0002) (Fig. 2a). However, blood loss was markedly reduced in cases treated by LMLR (WMD = − 107.4 ml, 95%CI − 179.0 to − 35.7, *P* = 0.003) (Fig. 2b). The other perioperative outcomes, i.e., blood transfusion (OR = 0.71, 95%CI − 0.34 to 1.49, *P* = 0.36) and resection margin (WMD = 0, 95%CI − 0.43 to 0.44, *P* = 0.98) rates were comparable in the LMLR and OMLR groups (Fig. 2c & d).

### Postoperative outcomes

**Fig. 3:**
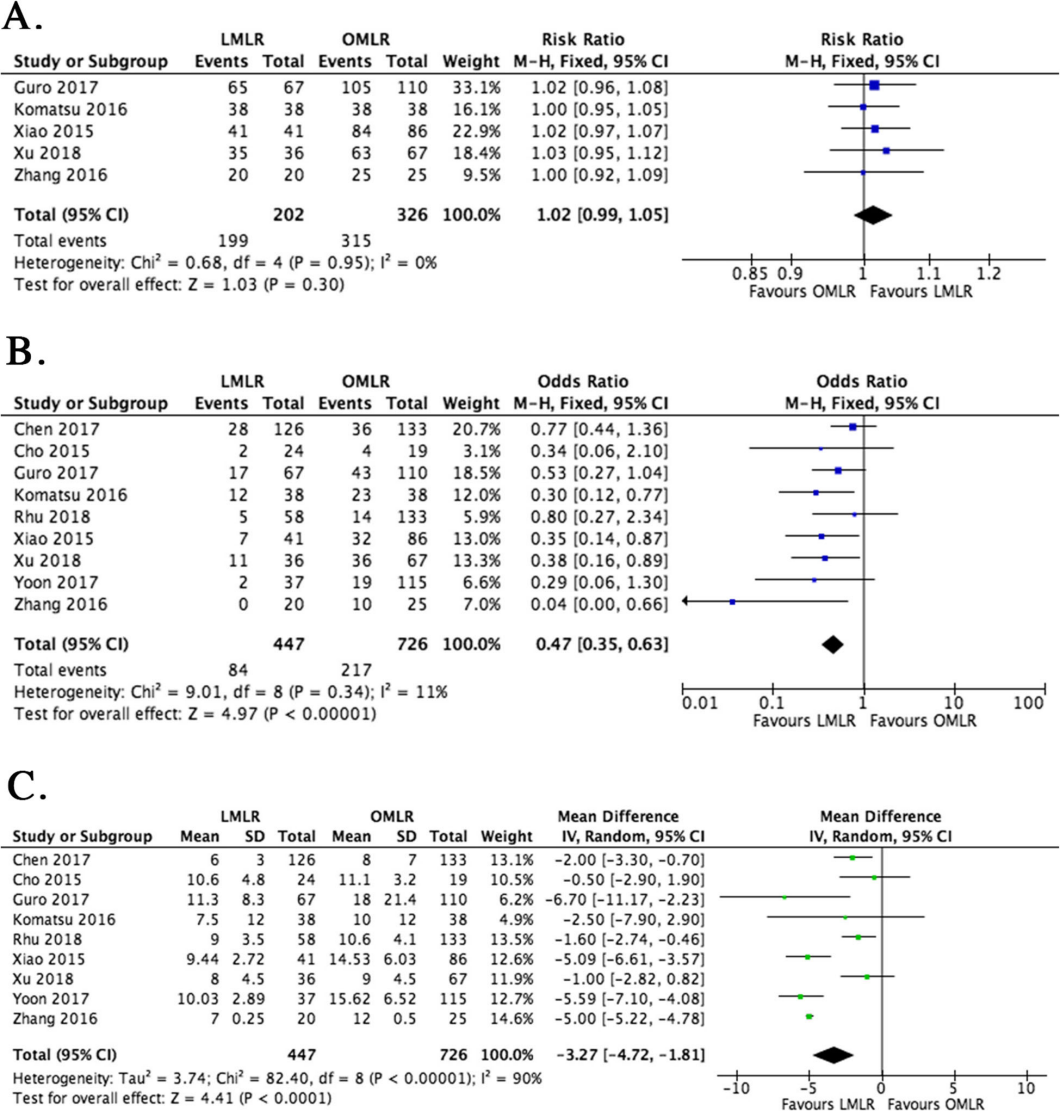
Forest plot depicting postoperative outcomes of LMLR versus OMLR. **a**. R0 resection in LMLR versus OMLR; **b**. Postoperative morbidity in LMLR versus OMLR. **c**. Hospital stay in LMLR versus OMLR. Weighted mean differences (WMDs) and Odds ratios (ORs) are shown with 95% confidence intervals (CIs). LMLR, laparoscopic major liver resection; OMLR, open major liver resection

The postoperative outcomes were summarized as follows. R0 resection rates were comparable in the LMLR and OMLR groups (OR = 1.02, 95%CI 0.99 to 1.05, *P* = 0.30) (Fig. 3a). LMLR treated cases showed markedly reduced morbidity postoperatively. The pooled OR for LMLR was 0.47 versus OMLR (95% CI 0.35 to 0.63, *P* <  0.0001) (Fig. 3b). The severities of these postsurgical morbidities are listed in Table 3. Hospital stay was reduced after LMLR by 3.27 days (95% CI − 4.72 to − 1.81 d, *P* <  0.0001), although the data were highly heterogeneous (I^2^ = 90%, *P* < 0.01) (Fig. 3c).

**Table 3:** Severity of the Complications

Reference (year)	Severity	n (%)		*P*
		LMLR	OMLR	
Cho JY (2015) [23]	NA	NA	NA	NA
Xiao L (2015) [24]	≤II	5 (12.2%)	25 (29.1%)	0.036
	≥III	2 (4.9%)	7 (8.1%)	0.764
Komatsu S (2016) [25]	≤II	7 (18.4%)	16 (42.1%)	0.023
	≥III	5 (13.2%)	7 (18.4)	0.529
Zhang Y (2016) [26]	I	20 (100%)	15 (60%)	< 0.05
	II	0 (0)	8 (32%)	< 0.05
	III	0 (0)	2 (8%)	< 0.05
Chen JH (2017) [27]	II	14 (11.1%)	24 (18.0%)	0.115
	III	0 (0)	4 (3.0%)	0.123
	IV	2 (1.6%)	4 (3.0%)	0.685
Yoon YI (2017) [28]	NA	NA	NA	NA
Guro H (2018) [29]	≤II	10 ()	16 ()	0.029
	≥III	7 ()	27 ()	
Rhu J (2018) [30]	≤II	4 (6.9%)	9 (6.8%)	0.528
	≥III	1 (1.7%)	2 (1.5%)	
Xu H (2018) [31]	≤II	11 (30.6%)	24 (35.8%)	0.024
	≥III	0 (0)	12 (17.9%)	0.017

Variables are expressed as no. (%). NA, not available. Abbreviations: LMLR, laparoscopic major liver resection; OMLR, open major liver resection

### Long-term oncological outcomes

**Fig. 4:**
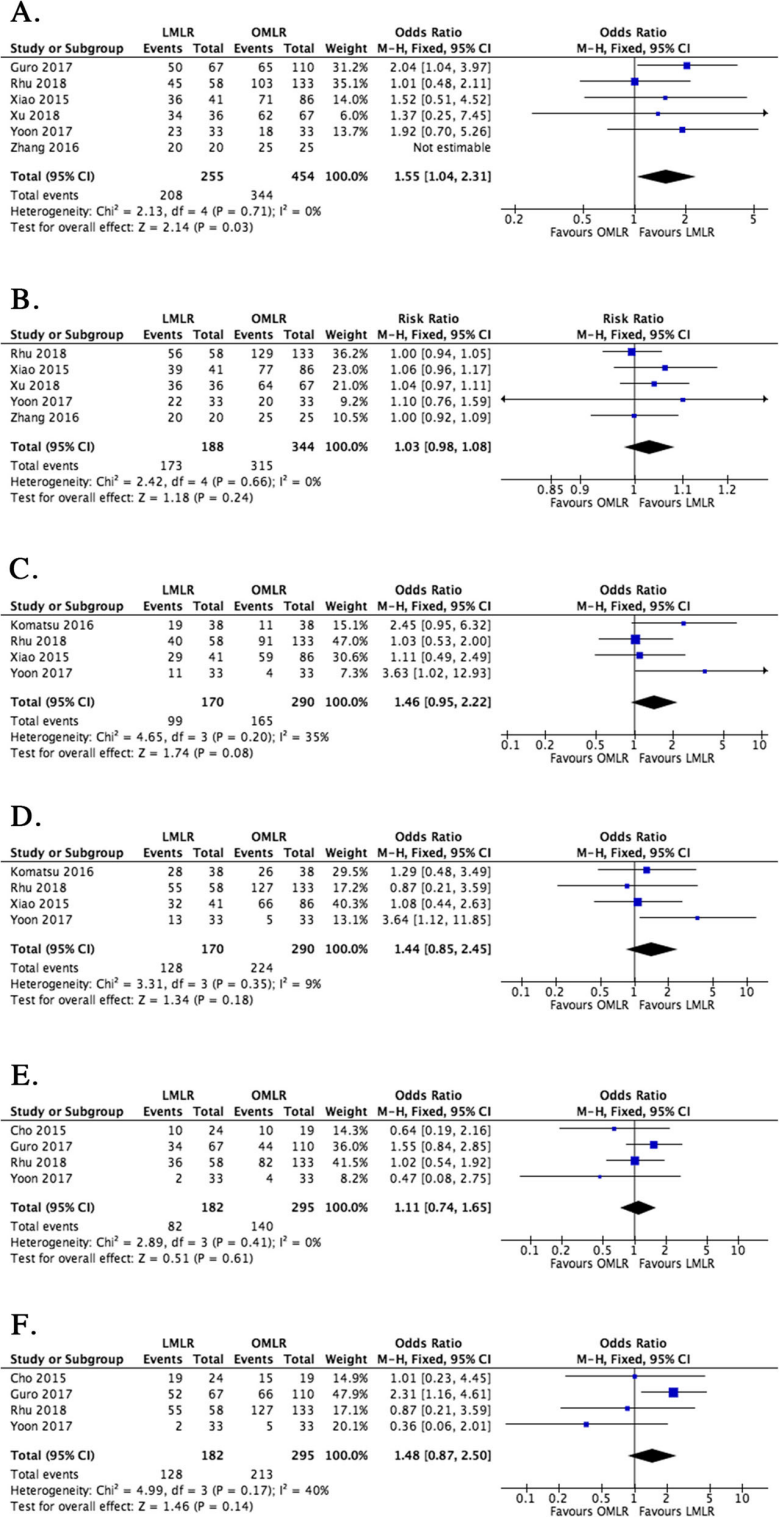
Forest plots depicting the oncological outcomes of LMLR versus OMLR. **a**. 1-year disease-free survival (DFS) in LMLR versus OMLR; **b**. 1-year overall survival (OS) in LMLR versus OMLR; **c**. 3-year disease-free survival (DFS) in LMLR versus OMLR; **d**. 3-year overall survival (OS) in LMLR versus OMLR; **e**. 5-year disease-free survival (DFS) in LMLR versus OMLR; **f**. 5-year overall survival (OS) in LMLR versus OMLR. Odds ratios (ORs) are shown with 95% confidence intervals (CIs). LMLR, laparoscopic major liver resection; OMLR, open major liver resection

Although only 6 studies reported 1-year OS and DFS data, the results showed that 1-year DFS following LMLR was significantly improved compared with the OMLR group (OR = 1.55, 95% CI 1.04 to 2.31, *P* = 0.03). However, 1-year OS showed comparable values in the LMLR and OMLR groups (OR = 1.03, 95% CI 0.98 to 1.08, *P* = 0.24) (Fig. 4a & b). The 3-year and 5-year oncological outcomes (DFS and OS) showed no marked differences between the LMLR and OMLR groups (OR = 1.46, 95% CI 0.95 to 2.22, *P* = 0.08; OR = 1.44, 95% CI 0.85 to 2.45, *P* = 0.18; OR = 1.11, 95% CI 0.74 to 1.65, *P* = 0.61; OR = 1.48, 95% CI 0.87 to 2.50, *P* = 0.14) (Fig. 4c–f).

## Discussion

The expanding range of LLR procedures, from non-anatomic wedge-, left lateral-, and anterior hepatic segment resections to sectionectomy, hemihepatectomy, trisectionectomy, and resection of difficult posterior segments, is regarded as mimicking OLR expansion [4, 10]. This expansion of LLR procedures is associated with both technological (instruments) and technical (skills) advances. In this period, two international consensus conferences have summarized the current status and future perspectives of LLR [9, 10]. Although multi-center and prospective, randomized studies would be ideal for assessing the effectiveness and safety of LMLR versus OMLR, an increasing amount of studies evaluating LMLR have been reported since 2009. However, minor resections constitute the vast majority of procedures in clinical practice. LMLR remains limited to very few centers and requires further evaluation and caution [4, 8, 15].

LLR has a particularly critical function in HCC treatment [32]. However, due to technical difficulties and the unique anatomical features of the liver, LLR remains somehow limited to a few high-volume and specialized centers [33, 34]. Berardi et al. reported a cohort study assessing perioperative and oncological outcomes from 4 European specialized centers [34]. The study revealed that the percentage of liver resections in which laparoscopy was applied yearly had increased from 5 to 43% during the past 15 years. They also found that perioperative and oncological outcomes have improved significantly with time and reached a stable level in the last few years [34]. However, HCC is commonly associated with chronic liver disease, cirrhosis, and/or impaired liver function, which might increase the risk of severe morbidity postoperatively and decrease the long-term survival rate [35]. Over the past quarter-century, there have been a number of studies evaluating perioperative and/or oncological outcomes of the LLR treatment in HCC patients. The laparoscopic technique and surgical care have been improved to establish LLR standardization [4, 6, 34]. These advances have remarkably increased the application of laparoscopic major liver resection (LMLR) in the last 10 years [6, 15, 31].

Studies or meta-analyses comparing laparoscopic methods to OLR all reported decreased blood loss, lower transfusion rate, reduced post-surgical morbidity, and decreased hospital stay, with comparable oncological outcomes [15, 35, 36]. However, the majority of trials focused on minor liver resection or failed to clearly differentiate between the outcomes of minor and major resections. Although the latest meta-analysis assessed short- and long-term outcomes between LMLR and OMLR, all retrospective trials comparatively evaluating LMLR and OMLR were included [37]. The combined results may be biased, so we conducted the current meta-analytical study of pooled perioperative and long-term LMLR and OMLR outcomes. Since no results of existing prospective randomized trials are currently available for analysis, totally 1173 patients from 9 retrospective trials were meta-analyzed. Our results demonstrated the technical feasibility and safety of LMLR in HCC patients. Our study included 9 published studies from major databases, comparing the short-term surgical and long-term oncological outcomes of LMLR and OMLR in the treatment of HCC. Only 9 studies were included in this meta-analysis because of the following plausible reasons. (1) We selected RCTs comparing LMLR with OMLR for HCC, and excluded studies reporting minor liver resection or with unavailable outcomes of major liver resection. (2) We included studies that analyzed the outcomes of LMLR in HCC, and excluded those in which LMLR was applied for recurrent HCC, hepatic metastatic cancer, simultaneous resection of the liver and other organs, or simultaneous resection of malignant and benign liver tumors. (3) We excluded studies that included less than 20 patients, considering the notion that studies reporting LMLR data in small samples might have limited reliability [15].

Application of LLR was rather delayed by technical challenges in keeping homeostasis at the transection plane and managing intraoperative bleeding from intrahepatic vessels [7, 13]. Intraoperative bleeding remains one of the most challenging issues in LLR, especially when major liver resection is performed in HCC complicated with chronic liver diseases or cirrhosis. Therefore, bleeding during LLR remains one of the most common reasons for selecting OLR. As shown previously, blood loss and perioperative blood transfusion negatively affect short-term surgical and long-term oncological outcomes [26, 27, 34, 38, 39]. In order to decrease bleeding and perioperative blood transfusion, some surgical techniques, such as the Glissonian approach, anatomic liver resection and selective clamping, have been proposed, which might exert reduced deleterious effects on postoperative liver function and yield more positive outcomes [40–43]. Moreover, innovative methods, e.g. intraoperative ultrasonography, microwave-based coagulation, ultrasonic dissection, and argon beam coagulation, and the use of laparoscopic coagulation shears and endoscopic linear staplers, significantly help achieve appropriate homeostasis in LLR [17, 44]. In this study, although the number of patients that required blood transfusion was not significantly lower in the LMLR group, the volume of blood loss was markedly reduced, suggesting bleeding control could be well conducted in LMLR. Considering other intraoperative outcome measurements, the operative time was markedly prolonged after LMLR. These results were consistent with those reported by Laurent et al [45] The longer operation time may mainly be attributed to the “learning curve” effect, complexity and wide resection plane in LMLR [23, 36]. Despite longer operation duration and the use of special laparoscopic equipment in the LMLR group, the patients had markedly reduced blood loss and hospitalization duration. In this study we did not investigate whether the benefits were cost-effective. A retrospective analysis showed that laparoscopic major liver resection exhibits a high potential clinical outcome effect compared with open major liver resection with cost-effectiveness [46]. However, we expect a future randomized trial to assess the benefits and costs of both surgical methods.

In this study, the pooled data showed that postoperative morbidity rates were markedly reduced after LMLR compared with OMLR. Although Nomi et al. reported a total of 183 cases that underwent LMLR and confirmed that postoperative morbidity was comparable in both LMLR and OMLR groups, only 28 cases of OMLR were included in the study [47]. Takahara et al. published the data of a national clinical database in Japan, with postoperative morbidity comparable to that described in this meta-analysis [7]. Complication severity was assessed using the modified Clavien classification in most of the included studies (Table 3). Although the severity of complications following LMLR showed an increasing trend compared with the OMLR group, other studies showed that severity was similar in both groups [45, 48]. Given the retrospective nature of the included trials, it was difficult to review more detailed data of complications to obtain more meaningful results.

The risk of inadequate resection margin, potential risk of tumor seeding, et al., were the main concerns regarding LLR use for HCC treatment [15]. However, the application of anatomic resection and ultrasound scanning during laparoscopic liver resection could help delineate the cancerous lesions, achieving the intended margin. At the same time, the improvement of laparoscopic technology and the available equipment for reducing potential tumor seeding such as plastic bags for specimen removal, may help overcome all these limitations [49]. Although a meta-analysis conducted by Lin et al. [15], confirmed no differences in oncological outcomes associated with laparoscopic and open minor liver resections for liver cancer, we still expect future trials to explore any differences in long-term oncological outcomes between LMLR and OMLR for HCC. As shown above, although 1-year DFS was elevated after treatment by LMLR compared with OMLR, oncological outcomes were comparable in both groups. Guro et al. reported markedly higher recurrence rate within 1 year of OMLR compared with LMLR (40.0% versus 25.8%, *P* = 0.029) [29], but the other included trials found no marked differences in 1-year DFS between these two groups. Although long-term oncological outcomes are not better with the laparoscopic method, some studies showed that unexpected diagnosis of early HCC could only be achieved by laparoscopy [50]. In addition, trials assessing HCC only in patients with chronic liver diseases also demonstrated equivalent OS and the DFS after LMLR and OMLR, which suggests that the tumor recurrence rate for the liver parenchyma (or other tissues) is not elevated after LMLR. This is consistent with the results of the current meta-analysis.

The major shortcoming of the current report was that only retrospective non-randomized controlled trials were included for review and meta-analysis. Therefore, it was difficult to review enough data and information to obtain meaningful results. In most of the included studies, cases were assigned to either the LMLR or OMLR group according to their preoperative clinical data and tumor characteristics, so selection bias was inevitable. However, three included studies used the propensity score matching method to minimize bias [28, 30, 31]. The propensity score matching method is considered one of the most optimal tools for reducing selection bias in non-randomized studies [51, 52]. In addition, by focusing only on major liver resection, we might have missed a broader group of studies in which patients undergoing major liver resection represented only a subset of the entire population. However, the available data regarding major liver resection in these studies were very difficult to assess. In order to obtain additional relevant studies, we extended our search to “related articles,” and manually searched interesting references listed in the retrieved articles. Last but not the least, the small sample sizes of multiple trials also reduced data reliability. Although the methods recommended by Hozo and colleagues are mostly acceptable, they constituted a limitation in the present meta-analysis, mainly because the most important aspects of the analysis involved continuous variables and WMDs [18].

However, the data available from the included studies were published by high-volume and specialized centers that could perform LMLR as well as OMLR. At the same time, strict eligibility criteria were used to ensure the quality of included studies upon extensive search of the available literature. The Meta-analysis of observational studies in epidemiology (MOOSE) guidelines recommended by Stroup et al. [18] and the NOS were used for assessing study quality and risk of bias, and publication bias was minimal. Furthermore, the timing of this meta-analysis was inadequate since the global use of LMLR has increased dramatically in the past 10 years, as well as the amount of available data on LMLR and OMLR in HCC.

## Conclusions

Following a meta-analysis of comparative cohort trials, our results revealed that in comparison with OMLR, LMLR may cause reduced bleeding, decreased postoperative morbidity, and shorter hospitalization in HCC; however, LMLR had prolonged operative time. The long-term oncological outcomes assessed were comparable in both groups. Retrospective studies and the small sample sizes of several included studies may decrease the reliability of these results. Therefore, large multi-center, prospective randomized trials are required to further assess the surgical and oncological outcomes of LMLR versus OMLR.

## Data Availability

The datasets used and/or analyzed in the current study are available from the corresponding author on reasonable request.

## Abbreviations

CI: Confidence interval
DFS: Disease-free survival
HCC: Hepatocellular carcinoma
LLR: Laparoscopic liver resection
LMLR: Laparoscopic major liver resection
MOOSE: Meta-analysis of observational studies in epidemiology
NOS: Newcastle–Ottawa scale
OLR: Open liver resection
OMLR: Open major liver resection
OR: Odds ratio
OS: Overall survival
WMD: Weighted mean difference
